# Processing of auditory information in forebrain regions after hearing loss in adulthood: Behavioral and electrophysiological studies in a rat model

**DOI:** 10.3389/fnins.2022.966568

**Published:** 2022-11-10

**Authors:** Marie Johne, Simeon O. A. Helgers, Mesbah Alam, Jonas Jelinek, Peter Hubka, Joachim K. Krauss, Verena Scheper, Andrej Kral, Kerstin Schwabe

**Affiliations:** ^1^Department of Neurosurgery, Hannover Medical School, Hanover, Germany; ^2^Cluster of Excellence Hearing4all, German Research Foundation, Hanover, Germany; ^3^Hannover Medical School, Institute of Audioneurotechnology, Hanover, Germany; ^4^Department of Experimental Otology of the ENT Clinics, Hannover Medical School, Hanover, Germany; ^5^Department of Otolaryngology, Hannover Medical School, Hanover, Germany

**Keywords:** deafness, auditory brainstem response, cognition, learning, memory, single unit activity, local field potential, hearing loss

## Abstract

**Background:**

Hearing loss was proposed as a factor affecting development of cognitive impairment in elderly. Deficits cannot be explained primarily by dysfunctional neuronal networks within the central auditory system. We here tested the impact of hearing loss in adult rats on motor, social, and cognitive function. Furthermore, potential changes in the neuronal activity in the medial prefrontal cortex (mPFC) and the inferior colliculus (IC) were evaluated.

**Materials and methods:**

In adult male Sprague Dawley rats hearing loss was induced under general anesthesia with intracochlear injection of neomycin. Sham-operated and naive rats served as controls. Postsurgical acoustically evoked auditory brainstem response (ABR)-measurements verified hearing loss after intracochlear neomycin-injection, respectively, intact hearing in sham-operated and naive controls. In intervals of 8 weeks and up to 12 months after surgery rats were tested for locomotor activity (open field) and coordination (Rotarod), for social interaction and preference, and for learning and memory (4-arms baited 8-arms radial maze test). In a final setting, electrophysiological recordings were performed in the mPFC and the IC.

**Results:**

Locomotor activity did not differ between deaf and control rats, whereas motor coordination on the Rotarod was disturbed in deaf rats (*P* < 0.05). Learning the concept of the radial maze test was initially disturbed in deaf rats (*P* < 0.05), whereas retesting every 8 weeks did not show long-term memory deficits. Social interaction and preference was also not affected by hearing loss. Final electrophysiological recordings in anesthetized rats revealed reduced firing rates, enhanced irregular firing, and reduced oscillatory theta band activity (4–8 Hz) in the mPFC of deaf rats as compared to controls (*P* < 0.05). In the IC, reduced oscillatory theta (4–8 Hz) and gamma (30–100 Hz) band activity was found in deaf rats (*P* < 0.05).

**Conclusion:**

Minor and transient behavioral deficits do not confirm direct impact of long-term hearing loss on cognitive function in rats. However, the altered neuronal activities in the mPFC and IC after hearing loss indicate effects on neuronal networks in and outside the central auditory system with potential consequences on cognitive function.

## Introduction

Hearing loss is one of the most frequent sensory deficits affecting more than 5% of the worldwide population (World Health Organization, 2012). In elderly people, it is strongly associated with dementia (Lin et al., 2013; Lin and Albert, 2014; Kral et al., 2016; Livingston et al., 2020). It also negatively affects social communication (Maharani et al., 2019), which may contribute to cognitive decline. The concept that hearing and cognitive functions are tightly linked and interdependent is also indicated by the fact that speech recognition outcomes of cochlear implant patients depend on the cognitive abilities of the recipient (Akeroyd, 2008; Lazard et al., 2012; Naples et al., 2021). With that regard, it has been proposed that treatment of hearing loss may prevent the development of neuronal dysfunction and cognitive decline after hearing loss (Naples et al., 2021; Powell et al., 2021). Recent studies also propose that successful hearing rehabilitation, including cochlear implants, may positively affect cognitive abilities and neuropsychiatric symptoms (Mamo et al., 2018; Mosnier et al., 2018; Kim et al., 2021). Nevertheless, whether hearing loss and cognitive decline with age simply share common risk factors, or whether there is a causal link, is not yet known (Nadhimi and Llano, 2021).

Animal models offer the opportunity to investigate the causality between hearing loss and cognitive decline in a confined setting, especially in the absence of language, which may act as a confounding factor. Numerous studies investigated the effect of hearing loss on auditory function and neural plasticity throughout the auditory pathway (Cardon et al., 2012; Wingfield and Peelle, 2015). The impact of hearing loss on non-auditory function, however, has been primarily addressed with regard to hippocampal function (reviewed in Nadhimi and Llano, 2021). Nevertheless, consequences of hearing loss on executive functions and brain regions subserving executive function, such as the prefrontal cortex (PFC), have been suggested (Galloway et al., 2008; Ball et al., 2011; Kronenberger et al., 2013, 2014; Kral et al., 2016; Marschark et al., 2017). In this context, also the inferior colliculus (IC) of the auditory pathway is interesting, since this region is highly interconnected with sensory, motor, and cognitive networks (Gruters and Groh, 2012; Melo-Thomas and Thomas, 2015; Olthof et al., 2019; Cauzzo et al., 2022).

The PFC is a key area for the regulation of higher brain functions. It is involved in various cognitive processes with higher-level stages of information processing, as needed in the categorization and recognition of prosodic stimuli (Brück et al., 2011). In order to perform such higher-order processing, the PFC is connected to several limbic brain areas, such as the hippocampus, the amygdala, the nucleus accumbens, and the ventral tegmental area (Russo and Nestler, 2013; Li et al., 2015). A recent neuroimaging study on humans with long-term hearing loss reported a higher coupling between auditory areas and the dorsolateral PFC (Luan et al., 2019), the putative homologue to the rodent medial PFC (reviewed in Laubach et al., 2018).

We here aimed to examine a possible causal relationship between hearing loss and cognitive impairments in an animal model with defined hearing loss, and to identify possible neural correlates within the auditory system and the PFC. Hearing loss was induced with intracochlear injection of neomycin in adult rats. Thereafter, all rats were regularly tested for social behavior and motor function up to 1 year after deafening. Cognitive behavior after hearing loss was tested in the 4-arms baited 8-arms radial maze test, starting 8 weeks after surgery to allow plastic neuronal changes after deafening to occur. With this paradigm different aspects of cognitive behavior can be addressed (a) procedural memory, i.e., knowledge that some of the arms are baited and that food can be obtained from a baited arm only once during each training trial. In order to reach a certain criterion of success (i.e., visiting only baited arms with only minor number of errors), rats usually need to be trained for 3–4 weeks with 6 runs per day. At this point, (b) reference memory, i.e., remembering, which arms had been baited before, can be challenged by introducing days or even weeks between testing. Additionally, once the rats have learned the paradigm, cognitive flexibility (c) can be tested by baiting arms that have never been baited for a certain rat before, which is called “reversal learning”. One year after hearing loss, rats underwent electrophysiological recordings of single unit activity (SUA) and local field potentials (LFP) in the medial PFC (mPFC) and the IC of the central auditory pathway in a final setting. Within the mPFC, electrophysiological recordings were acquired at the prelimbic area, since this subarea is suggested to be involved in information processing for working memory and for spatial memory, as well as for the selection and maintenance of learning strategies (Oualian and Gisquet-Verrier, 2010; Casado-Román et al., 2020). Furthermore, coherence of these regions with the sensorimotor cortex (SMCtx) was analyzed. These experimental investigations will provide a fingerprint for cognitive impairment after hearing loss without language as potentially confounding factor.

## Materials and methods

### Animals

Adult male Sprague-Dawley rats (Charles River Laboratories; *n* = 26) were housed in groups of two to four animals per Makrolon Type IV open cages under controlled environmental conditions (22 ± 2°C; 55 ± 10% humidity; 10/14 h dark/light cycle with lights on at 06:00 a.m.). Tap water was freely available and standard rodent chow (Altromin, Lage, Germany) was fed with 14–16 g per day and animal to enhance saliency of reward pellets while ensuring weekly weight gain of about 5–15%. Body weight and clinical scores were assessed at least two times a week to ensure the well-being of the rats.

All experiments were carried out in accordance with the EU directive 2010/63 and were approved by the local animal ethics committee (Lower Saxony State Office for Consumer Protection and Food Safety, AZ 18/2874). All efforts were made to minimize the number of animals and their suffering.

### Study design

At age of 8–9 weeks (mean weight of 240 g), animals were randomly assigned to one of the three experimental groups: (1) deaf group (*n* = 12) where hearing loss was induced via intracochlear injection of neomycin, (2) sham group (*n* = 5) where the same surgical procedure was performed without opening the inner ear and without injection of neomycin, and (3) naive group (*n* = 9) which served as controls. Only rats of one experimental group were housed together. Acoustically evoked brainstem responses (ABR) measurements were conducted before surgery in all rats to ensure normal hearing; after surgery successful induction of bilateral hearing-loss was confirmed in the deaf group, respectively, normal hearing in the sham and naïve group by ABR measurements. At the end of the study, the hearing status of each animal was verified in all three groups with final ABR measurements. When first placed into the open field, exploratory behavior will play a major role for outcome and will also affect first time testing of social interaction, whereas during repeated testing rats already remember the open field. With regard to Rotarod testing, animals improve especially during first time exposure since animals learn to maintain balance (Carter et al., 1999; Crawley, 1999). Therefore, to familiarize rats to the test environments, all groups were exposed to open field, Rotarod, and social interaction 1 week before surgery. Thereafter, testing started just before surgery (Pre-surgery), which allowed testing for unintentional differences after random assignment of rats to the different experimental groups. Postoperatively, all rats were tested in weeks 1, 2, 4, 8, 16, 24, 32, 40, 48, and 56 after surgery.

**FIGURE 1:**
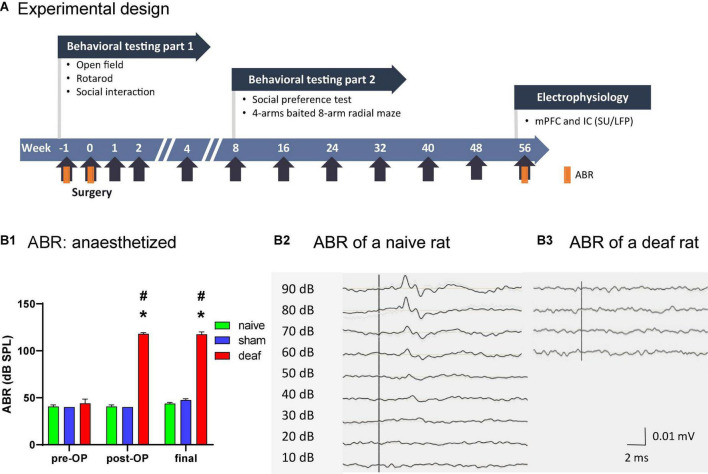
Timeline of the experimental design **(A)**. Effects of the deafening surgery to the auditory brainstem response of naïve, sham, and deaf rats with data shown as means ± SEM pre OP, post OP, and at final measurements 56 weeks after surgery. Differences between deaf rats and sham or naïve controls are shown as asterisks (*) and to pre-OP values as rhombus (^#^*P* < 0.05 after ANOVA; **B1**). Representative examples of ABRs of a naive **(B2)** and of a deaf rat **(B3)** are shown. ABR, auditory brainstem response. IC, inferior colliculus; LFP, local field potential; mPFC, medial prefrontal cortex; SU, single unit.

Extensive region- and layer-specific plasticity across the higher-order sensory cortices have been demonstrated 2 weeks postnoise exposure (e.g., Schormans et al., 2018; Chang et al., 2019). Nevertheless, other groups have started behavioral testing for long term effects of hearing loss on cognition 4–12 weeks after induced hearing loss (Liu et al., 2016; Wieczerzak et al., 2021). We therefore chose week 8 to start with testing of long term effects on cognitive behavior. From week 8 on, rats were tested for social preference. Training for high performance/success in the 4-arm baited 8-arm maze test also started in week 8, followed by retesting for performance in week 16, 24, 32, 40, 48, 56, and final testing for reversal learning. A summary of the experimental design is shown in Figure 1A. After behavioral testing at week 56, neuronal activity was recorded in the mPFC and the IC in a final setting. The correct placement of electrodes for the electrophysiological recordings was verified in Nissl-stained brain sections. Loss of hair cells in deaf rats, as well as their preservation in sham-deafened and naïve controls was exemplarily verified after histological processing of the cochleae.

### Surgery for deafening

All surgical procedures were performed under general chloral hydrate anesthesia (SIGMA; 360 mg/kg, i.p.), complemented by infiltration of the postauricular region with local anesthetic (Xylocain; 1% AstraZeneca, Wilmington, DE, USA) and systemic analgesia (5 mg/kg Carprofen, s.c., CP-Pharma, Burgdorf, Germany). Rats were placed in a lateral recumbent position, the skin incised for an approximate length of 3 cm, the muscles pulled caudally and the bulla exposed. A slow speed 0.5 mm diameter diamond burr was used to drill a small hole into the bulla to display the round window, the tympanic cavity, and the ossicles. For the administration of neomycin via the round window, 5 mg neomycin was dissolved in 1 ml of PBS (adjusted to pH 7.4). A lancet was used to perforate the round window and a 30-gauge needle and micro syringe (Hamilton) were used to inject 15 μl neomycin (Wefstaedt et al., 2006). The bullostomy was closed with dental cement and the skin was sutured. The same procedure was repeated for the second ear. Animals received systemic analgesia at the day of surgery and for two more days subsequently (2.5 mg/kg Carprofen s.c.).

### Hearing threshold measurements

ABR were recorded in all rats under general chloral hydrate anesthesia directly before and after the surgery as well as before the final electrophysiological measurements. ABR measurements were performed using subdermal needle electrodes, which were positioned at the vertex of the skull, behind the left or right ear and in the neck as grounding electrode. ABRs were obtained from vertex vs. retroauricular electrode by 40 dB preamplification (AMP55, Otoconsult Comp., Frankfurt, Germany) and 60 dB amplification (PNS1, Otoconsult Comp., Frankfurt, Germany). A second order bandpass filter (100–5,000 Hz) was used to eliminate low-frequency signals from the recordings.

Stimuli were generated by Audiology Lab stimulus generation and acquisition system (Otoconsult Comp., Frankfurt, Germany) running on a PC and connected to an AD converter (National Instruments NI-USB-6251, Austin, TX, USA) and an analog USB-controlled attenuator (PNS1, Otoconsult Comp., Frankfurt, Germany) connected to a calibrated DT48 speaker (Beyerdynamic, Heilbronn, Germany) positioned close to the ear. The clicks of 50 μs duration were presented at levels from 10 to 100 dB SPL p.e. in 10 dB steps in control and sham rats. After intracochlear injection of neomycine, however, rats were tested for ABR up to 120 dB to ensure complete hearing loss. The interstimulus interval was 113 ms and clicks were repeated 200 times for each stimulus level. The ABR signals were recorded using a hardware filter (100–5,000 Hz). For analysis, the recorded ABR signals were additionally digitally filtered (300–3,000 Hz) and averaged using custom codes in MATLAB (The MathWorks, Natick, MA, USA). ABRs were determined manually by detection of clearly identifiable waves II and III at latencies between 2 and 4 ms in ABR signal (Melcher et al., 1996; Tillein et al., 2012; Schopf et al., 2014).

### Behavioral testing

#### Motor behavior (open field, Rotarod) and social interaction

All rats were tested just before surgery (Pre-surgery), in weeks 1, 2, 4, 8, 16, 24, 32, 40, 48, and 56 after surgery. Behavioral testing of motor activity and interaction was done in a separate room with dim lighting. Two rats were first tested for motor activity in two open fields, thereafter placed into a third open filed for social interaction. Then, rats were tested consecutively on the Rotarod in another room with dim light.

##### Open field

Locomotive behavior was tested in an open field arena (60 cm × 60 cm × 30 cm). When first placed into the open field, exploratory behavior will play a major role for outcome whereas during repeated testing rats already remember the open field. Therefore, 1 week before testing started, rats were accustomed to the arena. For actual testing, rats were placed in the open field for 10 mins. The distance was automatically evaluated using video recordings from above the arena (TopScan; TopView Analyzing System 2.0; Clever Sys Inc., Reston, USA).

##### Rotarod

The effect of hearing loss on motor coordination and balance was assessed by placing the animals on a rotating rod inside a test chamber (10.5 cm × 43 cm × 43 cm; Rotarod, series 8, IITC Life Science). The rod rotated with accelerating speed for 60 s (starting speed: five rotations per minute; top speed: 15 rotations per minute) followed by another 60 s with constant speed (15 rotations per minute). One week before testing started, rats were trained to stay on the Rotarod. For actual testing, rats were placed on the rod for three consecutive trials and time was measured until the rat dropped of the rotating rod. For analysis, the mean latency of all three trials was calculated.

##### Social interaction

To evaluate if hearing loss affects the social interaction, two rats (cage mates of one experimental group) were placed into an empty open field arena for 10 mins. Social interactions were video recorded via a camera installed above the test arena. One week before testing started, rats were accustomed to being placed for social interaction in the open field. For analysis the duration of playing, following, anogenital, and frontal sniffing were evaluated. Total interaction duration was calculated as the sum of all evaluated behaviors.

#### Social preference and 4-arm baited 8-arm maze

Here testing started 8 weeks after surgery to allow plastic changes to neuronal network activity after deafening/sham-deafening before being tested. Rats were once tested for social preference on the day before each 8-arm maze session. Thereafter, 8-arm maze testing session was done in a separate room with full light.

##### Social preference

Testing in this paradigm started 8 weeks after surgery. After an acclimatization phase of 5 mins an object stimulus (empty wire mash cage) was introduced to the test rat. After additional 4 mins, the object stimulus was replaced by an identical cage filled with a male, age-matched, unfamiliar social interaction partner for another 4 mins (Ramos et al., 2016). The preference for the social or the object stimulus was evaluated using the duration of interactions with the respective stimuli.

##### 4-arm baited 8-arm maze

To investigate if the hearing loss has an effect on learning and memory, training in the radial 8-arm maze started 8 weeks after surgery. The maze consisted of eight arms (12 cm × 76 cm) equally distributed around a central platform (35 cm diameter) in 80 cm above floor level. At the far end of each arm a cup was installed that contained reward pellets (dustless precision pellets, 45 mg rodent purified diet, Bio-Serv, Flemington, NJ, USA). Training started with a habituation phase in which each animal was able to freely explore the whole maze.

Testing in the 4-arms baited 8-arms radial maze paradigm can be divided into three sections: (a) training rats to collect reward pellets from certain arms of the maze until reaching a certain criterion of success, which usually needs training the rats with 6 runs per day for 3–4 weeks, (b) retesting rats every 8 weeks. For final reversal learning (c), the previously baited arms were now unbaited (and vice versa) to test rat’s cognitive flexibility.

For training, four arms were randomly chosen for each individual rat (not more than two adjacent arms) and equipped with three reward pellets in each cup. Each run started by placing the animal facing a random direction in the center of the maze and was terminated if all rewards were collected by the animal or after 15 mins. On the first two training days the animals were tested with three consecutive runs (one block) and on the following days with six runs (two blocks). The sequence of entrances was tracked from outside the room via video recordings. For analysis, the number of entries in unrewarded arms [reference memory error (RME)] and the number of previously entered arms during the same run [working memory error (WME)] were assessed. The training was terminated upon “completion criterion” of less than three WME in three consecutive blocks of three runs, together with less than two RME per block.

After initial training for completion criterion, we focused on long-term memory by retesting rats in the radial maze test every 8 weeks. Here, rats were tested with six runs per day for two consecutive days (i.e., four blocks). Since some of the rats did not reach the initial training criterion, and consequently were disadvantaged at the following retesting, rats were retrained for criterion at the third (at week 32) and at the final retraining session (at week 56).

As final test all animals were subjected to a reversed training phase in which the reward pellets were placed in the previously unrewarded arms. Here rats were tested with six runs per day for two consecutive days (i.e., 4 blocks).

#### Electrophysiological recordings

For the extracellular SUA measurements and LFPs recording after final behavioral testing, rats were anesthetized with urethane (1.4 g/kg in 0.9% NaCl, i.p. ethyl carbamate, Sigma-Aldrich, St. Louis, USA); with additional doses as needed, depth of anesthesia was checked by the foot pinch. Rats anesthetized with urethane demonstrate spontaneous and cyclical alternations of brain state that resemble sleep state alternations or the global active or awake state of the brain. These fluctuations between different brain states are known as rapid eye movement (REM) and non-REM stages (Alam et al., 2017). Notably, during the REM state the brain metabolism is similar to normal waking values, is regarded more similar to the activity found in the awake condition, and was therefore used for analysis (Magill et al., 2000; Mallet et al., 2008; Rumpel et al., 2017).

The incision site was infiltrated with a local anesthetic, Xylocain. Rats were placed in a stereotaxic frame and body temperature was maintained at 37 ± 0.5°C by a heating device (FHC, Bowdoinham, ME, USA). Small craniotomies were made over the target coordinates for the mPFC and the IC. Thereafter the animal was placed in a Faraday cage to minimize electrical noise. A single microelectrode for extracellular recordings (quartz coated electrode with a platinum–tungsten alloy core (95–5%), diameter 80 μm, and impedance 1–2 MΩ) was connected to the Mini Matrix 2 channel version drives headstage (Thomas Recording GmbH, Gießen, Germany). The microelectrode signal was passed through a headstage with unit gain and then split to separately extract the SUA and the LFP components. For SUA recording, signals were band pass filtered between 500 and 5,000 Hz and amplified from 9,500 to 19,000 and sampled at 25 kHz. The stainless steel guide cannula, which touches the cortical surface, served as reference for the microelectrode (reported in Alam et al., 2012; Elle et al., 2020). Additionally, the ground wires were clamped on the neck and then plugged into the appropriate spot mini matrix head stage connector. The recordings in the mPFC and IC were stereotaxically guided at the following to the cortical surface; mPFC: anterior-posterior (AP) +3.2 and +2.7; mediolateral (ML) ±0.5 and ±0.8; ventral (V) –3.2 to –4.5; and for the IC: AP –8.7 and –9.1; ML ±1.3 and ±1.6; V –2.8 to –5.2.

A 1-mm-diameter jeweler’s screw which was positioned on the dura mater above the primary sensorimotor cortex (SMCtx; AP: –0.4 mm; ML: ±2.5 mm) served as electrode for recordings of the electrocorticograms (ECoGs). Additionally, a needle reference electrode was inserted in the neck muscles. The signal was filtered by using band pass (0.5–100 Hz) with a sampling rate of 1 kHz. Data were acquired using the CED 1401 A/D interface (Cambridge Electronic Design, Cambridge, United Kingdom). After termination of the experiment, electrical lesions were made at the recording sites to allow histological verification of the localization (10 μA for 10 s; both negative and positive polarities) as previously described (Jin et al., 2016).

##### Single unit analysis

Action potentials from single neurons were discriminated by the template-matching function of the spike-sorting software (Spike2; Cambridge Electronic Design, Cambridge, United Kingdom). Only well isolated SUs were included in the analysis, which was determined by the homogeneity of spike waveforms, the separation of the projections of spike waveforms onto principal components during spike sorting, and clear refractory periods in inter-spike interval (ISI) histograms. All analyses were performed using custom-written MATLAB (Mathworks, Natick, MA, USA) functions unless otherwise noted.

Firing rate (FR) was analyzed by taking the reciprocal value of the mean ISI for the whole 100 s of recording of spontaneous activity. The dispersion index (DI) of FR, a measure variance of firing rate was calculated by dividing the square of the standard deviation of the ISI by the mean ISI (std of ISI^2^/mean ISI) to compare the variation in their median values. A higher random firing pattern would be expected to have an increase in the diversity of the ISI lengths and higher DI of FR. A lower DI would be indicative of less diversity of the ISI lengths and more regular activity. The asymmetry index (AI) of firing was obtained by determining the ratio of the mode to the mean ISI, which provides information on the shape of the ISI histogram or the regularity of the discharge pattern. An asymmetry index close to 1 reveals a relatively regular firing pattern, whereas the more the index decreases from 1 the more irregular are the spike trains.

##### Local field potentials and coherence analysis

Representative epochs of 100 s without major artifacts were used for the frequency-domain signal processing for the LFPs in the mPFC, IC, and SMCtx-ECoGs. We visually checked for artifacts on the base of threshold for overshoot of amplitude and rejected affected epochs if necessary. The epochs were then converted in time domain spectrograms which is a time-varying spectral representation. Further, slow wave non-rapid eye movement (slow wave activity due to sleep) were identified by visual inspection and excluded. The spectrograms were computed by the squared magnitude of the short-time Fourier transform. The Fourier transformation was performed in each epoch and then averaged across all epochs. A finite impulse response 50-Hz notch filter and a 100-Hz low-pass filter were used. The spectral power of mPFC- and IC-LFPs, and SMCtx-ECoGs were derived by discrete Fourier transformation with blocks of 1,024 samples using a Welch periodogram in a custom Matlab script, which resulted in a frequency resolution of 0.9766 Hz. Hanning’s (referred to as Hann) window function was applied to control spectral leakage phenomenons. The relative power indices for each band were calculated from the absolute power in each frequency band and expressed as a percentage. For comparison of powers at different frequency bands, the area under the computed power density spectrum in specified frequency ranges for theta (4–8 Hz), alpha (8–12 Hz), beta (12–30 Hz), and gamma (30–100 Hz) frequency bands were calculated and averaged.

Further, the functional relationships between the SMCtx-ECoGs and LFPs of mPFC, and IC were estimated by means of coherence using methods described by Halliday et al. (1995). The SMCtx signals were recorded via a 1-mm-diameter jeweler’s screw electrode parallel with LFP recorded from the tip of the microelectrode either in the mPFC or in the IC. The coherence was analyzed from simultaneously parallel-recorded signals of either mPFC-LFP with SMCtx and IC-LFP with SMCtx. Coherence is one mathematical method of signal processing that can be used to determine the strength of oscillatory synchronizations across the brain networks in different neurological and neuropsychiatric disorders. High coherence between two brain areas represents higher synchronization and higher network connectivity. Coherence of oscillatory signals provides a frequency-domain measure of the linear phase and amplitude relationships between signals. It is a finite measure of values from 0 to 1, where 0 indicates that there is no linear association and 1 indicates a perfect linear association.

#### Histology

After electrophysiological recording, animals were euthanized and transcardially perfused with phosphate-buffered saline (PBS), followed by 4% paraformaldehyde in 0.1 m PBS. Brains were removed and postfixed in 4% paraformaldehyde followed by immersion in 30% sucrose for at least 24 h. Each brain was cut into 20-μm sections and stained with a standard hematoxylin and eosin (HE) protocol to verify the position of each electrode.

For histological verification of hearing loss, cochleae were prepared. Immersing with Spalteholz solution (five parts methyl salicylate and three parts benzyl benzoate; MSBB) allowed to take representative images of one deaf and one naïve control rat with a Leica TCS SP8 confocal laser scanning microscope using a 10× -objective [HC PL FLUOTAR 10×/0.30 DRY, Fa. Leica (Scheper et al., 2019)].

#### Statistics

Rats were first randomly assigned to the deaf, sham, or naïve group. Thereafter, preoperative measures for locomotor activity in open field, Rotarod and social interaction were compared to ensure that groups did not unintentionally differ before surgery for deafening or sham-deafening. Statistical analyses were performed using SigmaStat 4.0 software (Systat Software Inc., San Jose, CA, USA). For behavioral testing, two-way repeated measure analysis of variance (ANOVA) *F*-test followed by post hoc Bonferroni *t*-test for multiple comparisons with the factors group and repeated testing was used. For the comparisons of the electrophysiological data between control and the deaf group in either mPFC, IC, or SMCtx, a Mann-Whitney *U* test was applied to test for significant differences. The characteristics of the ISI histograms (mean, median, mode, and coefficient of variation) did not show normal distributions in most cases and therefore were analyzed with nonparametric multifactorial statistical methods. All tests were used two-sided; a *P* < 0.05 was considered significant.

## Results

### Outcome of deafening surgery

A total of 26 male normal hearing rats were used for the present experiments which were randomly assigned to *n* = 12 in the deaf group, *n* = 9 in the naïve group, and *n* = 5 in the sham group. After intracochlear neomycine injection, two animals were excluded from the deaf group because the ABR measurements showed insufficient success of the deafening surgery. These rats were entirely excluded from statistical analysis, leaving *n* = 10 rats as group size of the deaf rats. During the course of the experiments, two rats of the deaf group had to be sacrificed after testing in week 24, respectively, after testing in week 40 because of bad general condition not associated with the experiments, leaving *n* = 8 deaf rats for final behavioral testing. One animal of the sham group died during surgery, leaving a total of *n* = 13 animals for the control group (*n* = 9 from the naïve, *n* = 4 from the sham group). Moreover, one rat of the control group had to be sacrificed after testing in week 32, leaving *n* = 12 for final analysis.

### Hearing status over time

The click-evoked ABR thresholds before the deafening surgery did not differ between groups (mean over all groups: 42 dB SPL), whereas directly after the intracochlear injection of neomycin and at the final measurement 56 weeks after surgery the thresholds of the ABR measurements in the deaf group were significantly elevated to at least 100 dB. This was significantly enhanced both compared to the pre-OP time point (two-way ANOVA factor group *F*_1,40_ = 3668.7, *P* < 0.001; post-hoc testing, *P* < 0.05) and compared to the naïve and sham group (two-way ANOVA, interaction between factors group × week *F*_2,40_ = 140, *P* < 0.001; post-hoc testing, *P* < 0.05; Figure 1B1). The ABRs of the naive and sham group did not differ (Figure 1B1). Representative examples of ABR measurements of a naive (B2) and a deaf (B3) rat are shown in Figure 1.

Representative cochlea were stained to show hair cell loss after neomycin injection (Supplementary Figure 1). In naive rats, hair cells were preserved in the cochlea, whereas there was cell loss throughout the cochlea in the deaf rats (Supplementary Figure 1).

### Motor and social behavior

Since ABR and behavioral data did not differ between sham (*n* = 4) and naïve (*n* = 9) rats, both groups were combined as control group (*n* = 13) for further analysis. For analysis of open field, Rotarod, and social interaction by repeated measure ANOVA missing rat data were replaced by group mean. This affected one rat of the control group after week 32, as well as two rats of the deaf group after week 24, respectively, week 40 on).

#### Open field

**FIGURE 2:**
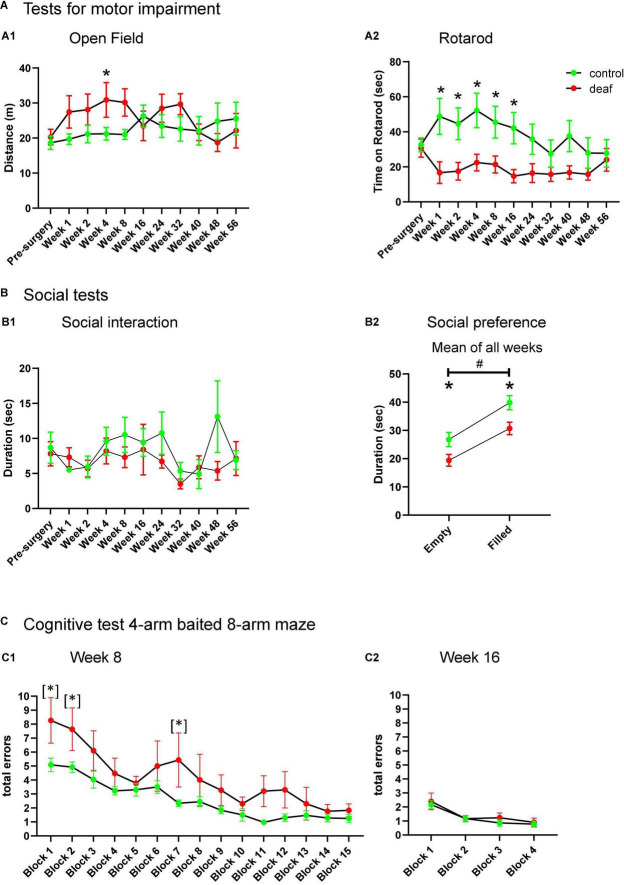
Performance of rats during testing for motor impairment **(A)**, social behavior **(B)**, and cognitive behavior **(C)** with data shown as means ± SEM for deaf and sham or naïve controls. Distance in open field **(A1)**, time on Rotarod **(A2)**, and time of social interaction **(B1)** is shown for the weeks of testing indicated with respect to measures just before surgery. The total duration of interaction with the empty containment versus containment filled with a social partner for all weeks of testing in the social preference paradigm is shown in panel **(B2)**. Total errors of rats initially learning the 4-arm baited 8-arm maze at week 8 after surgery for the different test blocks **(C1)** and during re-testing 16 weeks after surgery (i.e., 8 weeks after initial learning this paradigm, **C2**). Differences between deaf rats and controls are shown as asterisks (*P < 0.05 after ANOVA); asterisk in brackets indicate significant intergroup-differences after trend for significance in ANOVA (*). Differences between empty and filled containment are shown by hashtag (^#^*P* < 0.05 after ANOVA).

Control and deaf rats did not show differences in the total distance in the open field, except for week 4 after surgery, whereas rats of the deaf group covered more distance than those of the control group (two-way ANOVA factor group × week *F*_10,210_ = 2.010, *P* = 0.034; post-hoc testing, *P* < 0.05; Figure 2A1).

#### Rotarod

The Rotarod test showed a significantly decreased duration of the time on the Rotarod in deaf rats compared to control animals from week 1 to 16 after surgery (two-way ANOVA factor group × week *F*_10,210_ = 2.280, *P* = 0.015; post-hoc testing week 1–16 *P* < 0.05; see Figure 2A2), whereas from week 24 on groups did not differ (Figure 2A2).

#### Social interaction test

The total duration of interaction (sum of playing, following, anogenital, and frontal sniffing) did not differ between groups (Figure 2B1).

#### Social preference test

In the social preference test, deaf rats interacted less with either object, irrespective whether it was filled with a social partner or empty (Factor groups for “empty”: *F*_1,126_ = 4.685, *P* = 0.042, or “filled”: *F*_1,126_ = 7.028, *P* = 0.015; Supplementary Figure 3). To test whether the deaf group may interact less with the objects when filled with the social partner, we additionally applied repeated measure ANOVA with factor group and factor object (empty or filled). This two-way ANOVA showed a significant difference for the factor group (*F*_1,21_ = 7.19, *P* = 0.014) as well as for the factor empty/filled (*F*_1,21_ = 65.67, *P* < 0.001), whereas the interaction between factors was not significant (*F*_1,21_ = 0.351, *P* = 0.560). Post-hoc testing showed that deaf rats interacted less with the empty and the filled cage than the control group (*P* < 0.05). Both groups preferred interaction with the filled cage as compared to the empty cage (*P* < 0.05; Figure 2B2).

#### 4-arms baited 8-arm radial maze

One rat of the control group did not perform at all on the maze, and was therefore excluded from training, leaving *n* = 12 as control group. As the number of RME and WME during testing did not differ between groups in all sessions, we only report on analysis of total errors (sum of RME and WME). For statistical analysis of all radial maze settings blocks of three runs were used. During initial training for performance in the 4-arms baited 8-arm radial maze, starting week 8 after surgery, deaf rats made more total errors during training than the control group. Although ANOVA only showed a trend for significance for the factor group (*F*_1,280_ = 3.258, *P* = 0.086), we nevertheless performed post-hoc group comparisons, which shows significantly enhanced errors in blocks 1, 2, and 7 (all *P* < 0.05; Figure 2C1 showed as [*]). Retesting in weeks 16 (Figure 2C2), 24, 40, and 48 (Supplementary Figure 2) with four blocks also revealed no differences between deaf and control rats (Factor group “week 16”: *F*_1,60_ = 0.472, *P* = 0.499; Factor group “week 24”: *F*_1,60_ = 0.0158, *P* = 0.901; Factor group “week 40”: *F*_1,54_ = 0.102, *P* = 0.754; Factor group “week 48”: *F*_1,51_ = 0.0285, *P* = 0.868). Also retraining for performance at weeks 32 and 56 revealed no differences between groups (Factor group “week 32”: *F*_1,266_ = 0.1.355, *p* = 0.259; Factor group “week 56”: *F*_1,224_ = 0.042, *P* = 0.840). Finally, deaf and control rats did not differ during reversal training (Factor group “reversal training”: *F*_1,70_ = 0.291, *P* = 0.598; Supplementary Figure 2).

### Electrophysiology

In order to analyze functional status of the underlying neuronal structures, spontaneous SU activities were recorded from the mPFC and the IC. The total number of SUs recorded from the control group in them PFC was *n* = 617 and in the IC *n* = 394. In the deaf group, the total number of SUAs in the mPFC was *n* = 593, and in the IC *n* = 394, respectively.

**FIGURE 3:**
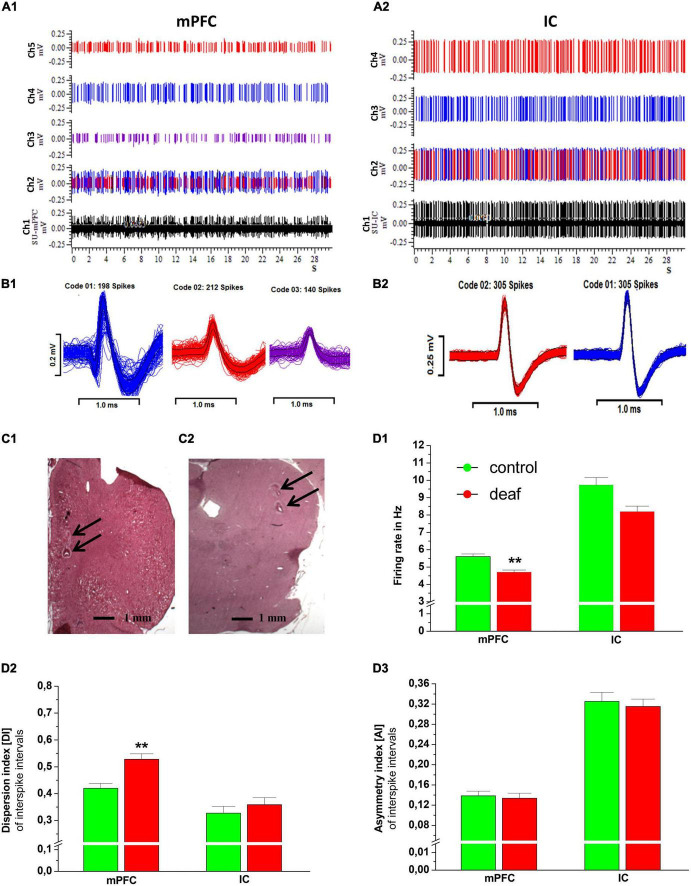
An example of 30 s recording epochs of the extracellular neuronal raw spikes from mPFC and IC are shown in panel **(A)** (channel 1 in black color). Channel 2 shows the raster plots with all different types of neuronal single units identified by spike sorting. Additionally, individual spikes of single units recorded in mPFC are shown in panel **(A1)** (channels 3, 4, and 5) and from IC in panel **(A2)** (channels 3 and 4). Corresponding action potential waveform of the mPFC and of the IC are shown in panels **(B1,B2)**. Representative examples of HE stained recording areas with electrical lesions (arrows) in coronal sections of the mPFC (RC from Bregma + 2.7; **C1**) and IC (RC from Bregma −8.7;**C2**). Firing rate **(D1)**, dispersion index **(D2)**, and asymmetry index **(D3)** for deaf and controls are shown as mean ± S.E.M. Differences between deaf rats and controls are shown as asterisks (*P < 0.05 after ANOVA).

The average SUs (mean and SEM) recorded per individual rat in the control group in the mPFC was 47.0 ± 5.4 and in the IC 32.83 ± 6.4. In the deaf group, the average recorded per individual rat for the mPFC was 65.8 ± 6.7 and for the IC 43.77 ± 9.26. An example of 30 s recording epochs of the extracellular single neuron spikes and its raster plots from the mPFC and the IC, together with action potential waveform of the mPFC and of the IC are shown in Figures 3A,B. Histology verified the correct position of the microelectrode used for the recordings in the mPFC and IC (Figures 3C1,C2).

### Single unit analysis

The Mann-Whitney *U* test showed that the FR in the mPFC was lower in the deaf group compared to the control group (*P* < 0.001), while in the IC this difference did not reach a level of significance (*P* = 0.11). The DI in the mPFC was higher in rats from the deaf group compared to the control group (*P* < 0.001). The DI of IC neurons did not differ between the control group and the deaf group. Furthermore, the AI of mPFC and IC neurons did not differ between the control group and the deaf group (Figure 3D).

### Local field potentials

For LFP and coherence analysis, we used neuron epochs from the mPFC control group (*n* = 176), the mPFC deaf group (*n* = 134), as well as from the IC control group (*n* = 128) and the IC deaf group (*n* = 128).

**FIGURE 4:**
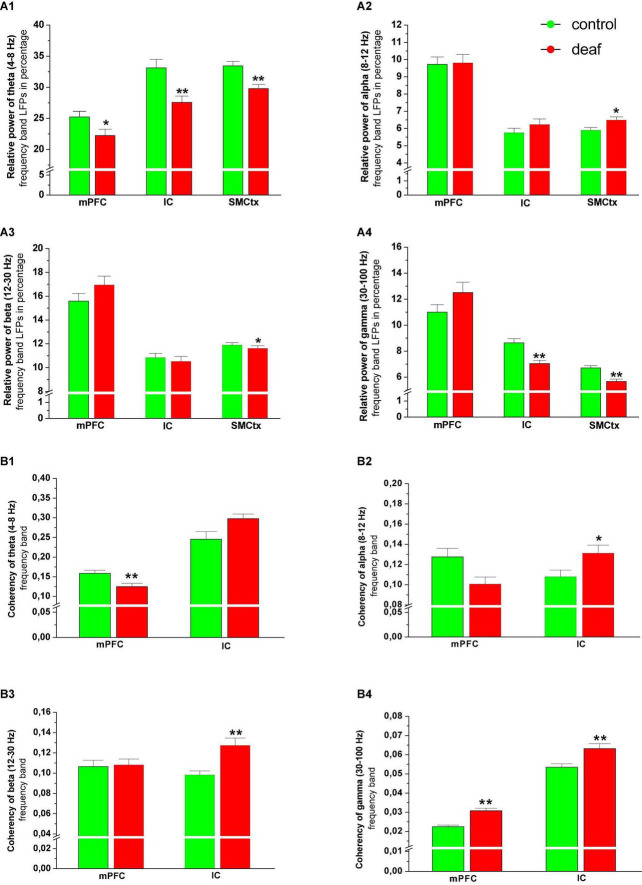
Relative power of theta **(A1)**, alpha **(A2)**, beta **(A3)**, and gamma **(A4)** oscillatory activity in the mPFC and IC area, as well as their coherence with SM-CtxECoG in the theta **(B1)**, alpha **(B2)**, beta **(B3)**, and gamma **(B4)** band shown as mean ± S.E.M. for deaf and sham or naïve controls. Differences between deaf rats and controls are shown as asterisks (*P < 0.05).

The Mann-Whitney *U* test revealed that the relative power of theta band activity was lower in deaf rats in all regions (mPFC: *P* < 0.05, IC area: *P* < 0.01, SMCtx-ECoG: *P* < 0.01). In the deaf group, alpha oscillatory activity was higher and beta oscillatory activity was lower only in the SMCtx-ECoG as compared to the control group (*P* < 0.05), while activity in mPFC and IC did not differ. For gamma oscillatory activity, analysis showed that the relative power was lower in rats from deaf group in the IC area (*P* < 0.01, SMCtx-ECoG: *P* < 0.01), while gamma activity in the mPFC did not differ (Figure 4A).

### Coherence with SMCtx-ECoG

Analysis of the coherence between the spontaneous LFP signals recorded from mPFC and SMCtx-ECoG showed that the theta frequency band coherency was reduced in the deaf group, while the gamma frequency band coherency was enhanced as compared to control groups (*P* < 0.01). For the IC and the SMCtx-ECoG the coherency for alpha (*P* < 0.05), beta (*P* < 0.01) and gamma (*P* < 0.01) was higher in the deaf group as compared to the control group (Figure 4B).

## Discussion

We here investigated whether deafening by local intracochlear injection of neomycin in adult rats would have long-term effects on motor function, non-auditory cognition, social interaction, and neuronal activity in brain regions associated with higher cognitive function.

Deaf rats had early learning deficits when training of the 4-arms baited 8-arms radial maze task started 2 months after surgery. Later during training, however, the number of working or reference memory errors did not differ between deaf rats and control groups. Re-testing every 2 months for up to 1 year, or final testing with reversal of baited arms also did not show differences between groups. These findings corroborate a recent study, where 30 days after noise-exposure rats with hearing deficits had difficulty in learning the initial rule of spatial paradigms, but only a tendency to not remember it during retesting (Wieczerzak et al., 2021). In mice, hearing loss induced by noise exposure also significantly decreased non-auditory memory function assessed with spatial learning and memory paradigms up to 3 months after exposure. These deficits correlated with the degree of hearing loss and were associated with synaptic changes in the hippocampus (Yu et al., 2011; Tao et al., 2015; Liu et al., 2016, 2018). However, effects were mainly found directly or within the first few weeks after noise-exposure, and often gradually recovered or were compensated over time (Haider et al., 2012; Uran et al., 2012). So far, only one group found that rats with severe hearing loss had deficits in the radial maze task and object recognition after 6 months (Park et al., 2016), albeit after 12 months these deficits disappeared (Park et al., 2018).

Together, while such data demonstrate effects of hearing loss on cognitive function and social interactions, the rodent data overall do not support the hypothesis that hearing loss by itself could induce a progressive cognitive decline (reviewed in Nadhimi and Llano, 2021). Compared with other findings in rodents about deficits in spatial learning paradigms, the effects observed in the present work after neomycin-induced hearing loss were rather mild. Stress related alterations (e.g., enhanced plasma corticosterone levels, increased hippocampal lipid peroxidation) which have been reported to occur after noise stress may also have contributed to hippocampal dysfunction and disturbed spatial learning (Manikandan et al., 2006; Gai et al., 2017; Hayes et al., 2019). The gradual improvement of performance in these tests may present recovery of early dysfunction or a compensatory mechanism.

In humans, it has been found that hearing loss increases the risk of social isolation and loneliness (Maharani et al., 2019), which also affects personal relationships and mental health (Manrique-Huarte et al., 2016). In the context of our experimental study, however, hearing loss did not affect social interaction, neither immediately after deafening, nor as a long-term consequence, which makes a causal relationship unlikely. In humans, communication by language is tightly linked and interdependent with social interaction (Weinstein and Ventry, 1982), while in rats – although they use ultrasound vocalization for nonspecific communication – “language” in its classical sense does not exist. Nevertheless, although rats did not differ in their preference for the social partner during the social preference test, rats with hearing loss were not as interested in a novel containment, irrespective of whether it was empty or it contained a social partner. Thus, rats with hearing loss may not be as curious about novel objects as controls, with consequences on learning the concept of cognitive paradigms, as seen in the initial phase of radial maze testing of the present study.

After neomycin induced hearing loss, rats were impaired on the Rotarod, a test traditionally used to assess motor deficits in rodents (Hamm et al., 1994; Brooks et al., 2012). In humans, hearing loss in children is associated with motor developmental deficits (Rajendran and Roy, 2011; Kamel et al., 2021), whereas motor function in adults with age-related hearing loss has not been extensively explored. However, reduced motor learning has been described in deaf adults performing a serial reaction-time task (Lévesque et al., 2014), which may be associated with cortical reorganization after age-related hearing loss. Authors speculated that nonauditory regions are upregulated to support speech perception after hearing loss, which may also contribute to the reorganization of resources with potential problems also with regard to cognitive and neural functioning (Slade et al., 2020). As a limitation one has to mention, that rats may learn to jump off by intention during repeated testing (Picciotto and Wickman, 1998). This will likely be the case in the control group, which stayed shorter on the Rotarod at the end of the experiments at week 24 and thereafter. Another limitation of this study may be repeated testing as well as testing all groups in different behavioral paradigms, which may have affected the outcome (Sousa et al., 2006; Bosch et al., 2022).

Despite only minor effects on behavior tested in this study, we found clear long-term effects of deafening on neuronal activity in the mPFC, a core structure for higher-order cognitive and executive functions (Teffer and Semendeferi, 2012). The prefrontal cortex is important for memory formation and retrieval with related executive roles in working memory, decision making, and cognitive flexibility (Cohen, 2011; Zielinski et al., 2020). In the mPFC of deaf rats, SUA was reduced and more irregular as compared to normal hearing controls. A similar pattern has been reported before in rat models for cognitive disturbances and neuropsychiatric disorders, where irregular firing or burst activity have even been considered more relevant for dysfunctional states than just considering mere changes in firing rates (Nambu et al., 2015). Interestingly, in the acute phase after deafening the activity of the mPFC did not differ from normal hearing controls, although the authors of that study already speculated that altered activity may manifest in the PFC at later time points due to long term plastic changes in the neuronal network (Wieczerzak et al., 2021).

In the present study the firing rate of the IC was reduced 1 year after deafening, whereas different groups reported on hyperexcitablity in regions of the central auditory pathway that develop within the first few weeks after hearing loss until up to 6 months after the trauma (Eggermont, 2015, 2017). However, although enhanced firing rates have been found after acoustic trauma (Mulders and Robertson, 2009), the firing rate was reduced 1–4 weeks after destroying cochlear input to the IC (Eggermont, 2015).

The auditory perception depends on the integration of multiple sensory and cognitive domains within the central auditory system as well as non-auditory regions. Nevertheless, the networks sub-serving this integration are unclear. Retrograde tracing studies in rats have shown that the IC of the auditory midbrain receives dense descending projections not only from the auditory cortex, but also from the visual, somatosensory, sensory, motor, and prefrontal cortices (Bajo and King, 2013; Olthof et al., 2019; Cauzzo et al., 2022).

Besides SUA, oscillations and rhythmic activities of functional neural networks are fundamental for complex perceptual and cognitive functions, including speech and social communications (Tendler and Wagner, 2015; Murphy and Benítez-Burraco, 2017), i.e., aspects that are affected in mental disorders, such as autism and schizophrenia (Jochaut et al., 2015; Yu et al., 2018). In the present study, the mPFC oscillatory theta band activity was reduced and gamma band activity was enhanced, also in coherence with the sensorimotor cortex. Altered theta band activity has also often been related to cognitive disturbances. In the mPFC, theta oscillations are modulated by spatial working memory and synchronize with the hippocampus through its ventral subregion (O’Neill et al., 2013). This is evident with complex cognitive functioning such as spatial memory, but also in the context of nonspatial learning (Zielinski et al., 2020). Altered theta band activity has also been described in psychiatric disorders characterized by cognitive disturbances. Together with reduced theta band activity, we demonstrated enhanced gamma band activity after hearing loss. Interestingly, during cognitive performance, especially in the context of spatial learning and memory formation, theta band activity between hippocampus and mPFC interacts with gamma band activity (Kupferschmidt and Gordon, 2018).

In the IC, theta and gamma band oscillatory activity was reduced, whereas coherence of alpha, beta, and gamma between IC and SMCtx was enhanced 12 months after deafening. How altered oscillatory activity may affect processes that contribute to speech processing has not been investigated, yet (Price et al., 2019).

It has been described that altered neuronal activity within frontal cortical areas may serve as a compensatory mechanism for deficits in speech processing in older adults, particularly in more challenging listening conditions such as hearing in noise (Binder et al., 2004; Zekveld et al., 2006; Du et al., 2016). Although with the non-auditory behavioral paradigms used in this study, we did not find major behavioral deficits or cognitive decline, neuronal recordings in the mPFC and IC demonstrated that hearing loss leads to long-term neuronal adaptations that are extensive and potentially detrimental for auditory processing and cognitive behavior not addressed in the present study (Harrison et al., 1998; Kral and Tillein, 2006; Kral and Sharma, 2012; Kral et al., 2019). Indeed, recent studies in auditory cortex demonstrate that top-down interactions in the auditory processing plays a key role for normal cortical processing (Yusuf et al., 2020). The PFC integrates manifold inputs to generate complex responses, such as decision making, cognitive flexibility, or the assessment of stimulus valence (Miller and Cohen, 2001; Vertes, 2006; Puig and Miller, 2015). With that regard, speech recognition not only depends on electrical stimulation of the cochlear implant and processing along the central auditory pathway, but also to processing in the PFC with high cognitive load (Winn, 2016). Our findings of long-term neuronal alterations in PFC and IC therefore support the clinical approach that hearing loss should be readily treated.

## Data availability statement

The original contributions presented in this study are included in the article/Supplementary material, further inquiries can be directed to the corresponding author.

## Ethics statement

This animal study was reviewed and approved by Lower Saxony State Office for Consumer Protection and Food Safety.

## Author contributions

MJ, SH, PH, JK, VS, AK, and KS: study concept. MJ, SH, MA, JJ, and KS: laboratory work and analyses. MJ and KS: scientific writing. All authors carefully revised the manuscript and approved the submitted version.

## Supplementary material

Supplementary Figure 1 Representative examples of a cochlea from a naive and a deaf rat. The arrows show preservation (naïve) vs. loss (deaf) of hair cells.

Supplementary Figure 2 Total errors of rats in the 4-arm baited 8-arm maze for re-training on weeks 24, 32, 40, 48, and 56, as well as for the reverse training in week 56. No differences were found between the deaf and control groups.

Supplementary Figure 3 Duration of interaction with the empty and the filled compartment during testing for social preference is shown as means ± SEM for deaf and sham or naïve controls for the weeks of testing. Differences between lesion rats and controls are shown as asterisks (**P* < 0.05).

## Abbreviations

AI: asymmetry index
ANOVA: analysis of variance
DI: dispersion index
ECoGs: electrocorticograms
FR: firing rate
HL: hearing loss
IC: inferior colliculus
ISI: inter-spike interval
LFPs: local field potentials
mPFC: medial prefrontal cortex
RME: reference memory error
SMCtx: primary sensorimotor cortex
SUs: single units
WME: working memory error.
