# Supplementary figures and images for: Processing of auditory information in forebrain regions after hearing loss in adulthood: Behavioral and electrophysiological studies in a rat model
Source: Front Neurosci. 2022 Nov 10;16:966568. doi: 10.3389/fnins.2022.966568 (PMC9684731; doi:10.3389/fnins.2022.966568)

## Slide 1
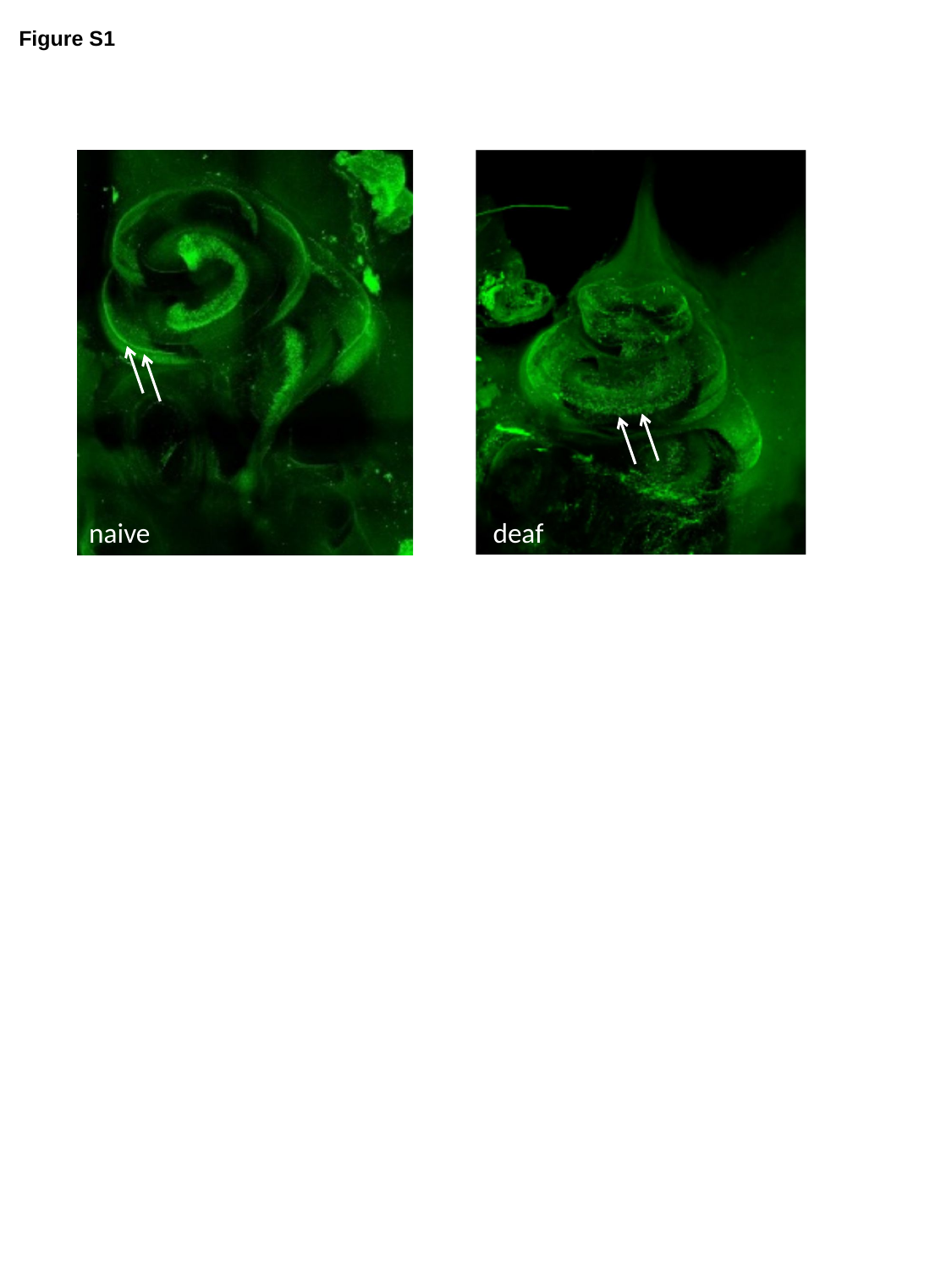

Figure S1
naive
deaf

Supplement: Supplementary Figure 1 — Representative examples of a cochlea from a naive and a deaf rat. The arrows show preservation (naïve) vs. loss (deaf) of hair cells. [file Presentation_1.PPTX]

## Slide 1
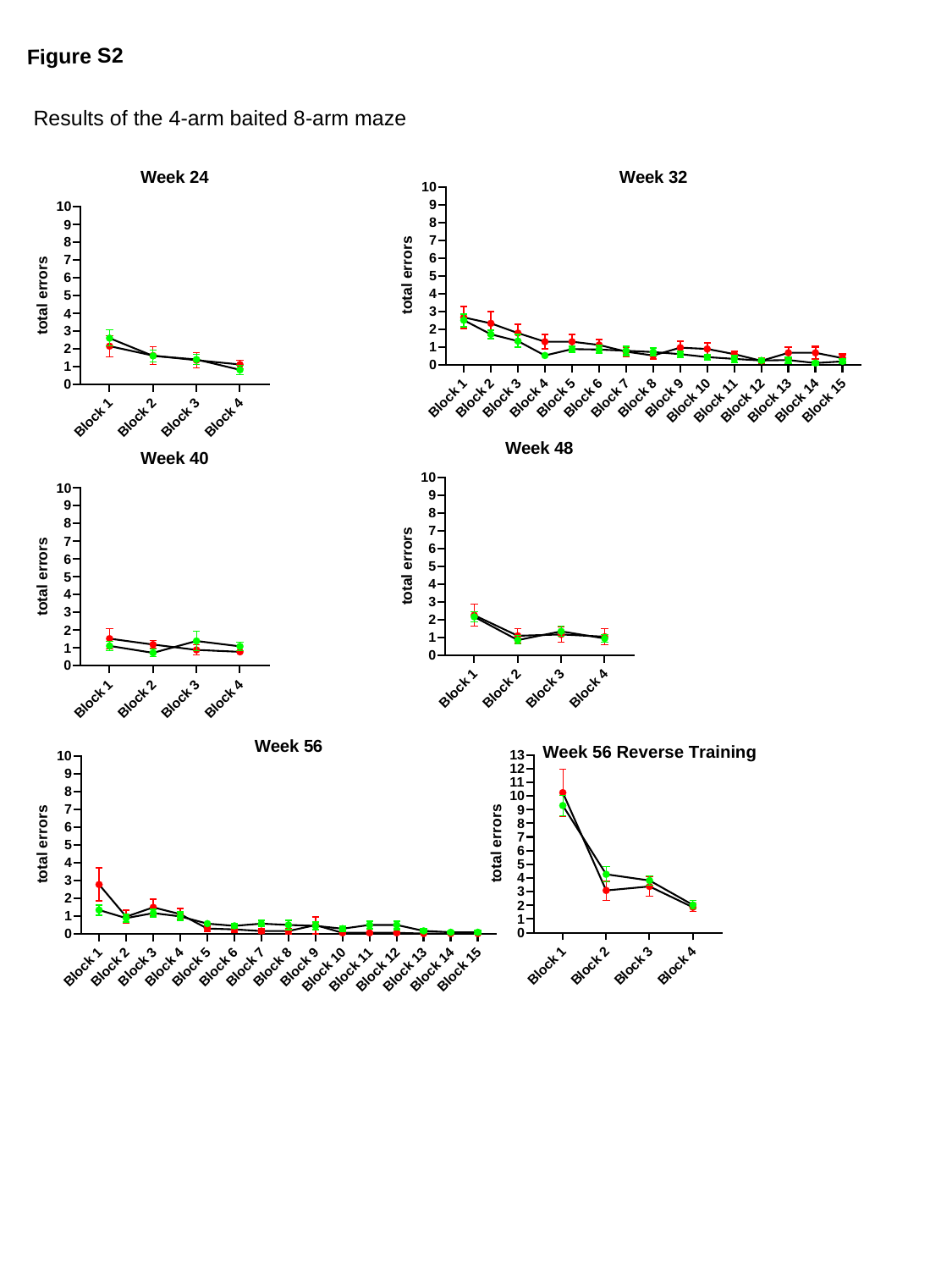

Figure S2
Results of the 4-arm baited 8-arm maze

Supplement: Supplementary Figure 2 — Total errors of rats in the 4-arm baited 8-arm maze for re-training on weeks 24, 32, 40, 48, and 56, as well as for the reverse training in week 56. No differences were found between the deaf and control groups. [file Presentation_2.PPTX]

## Slide 1
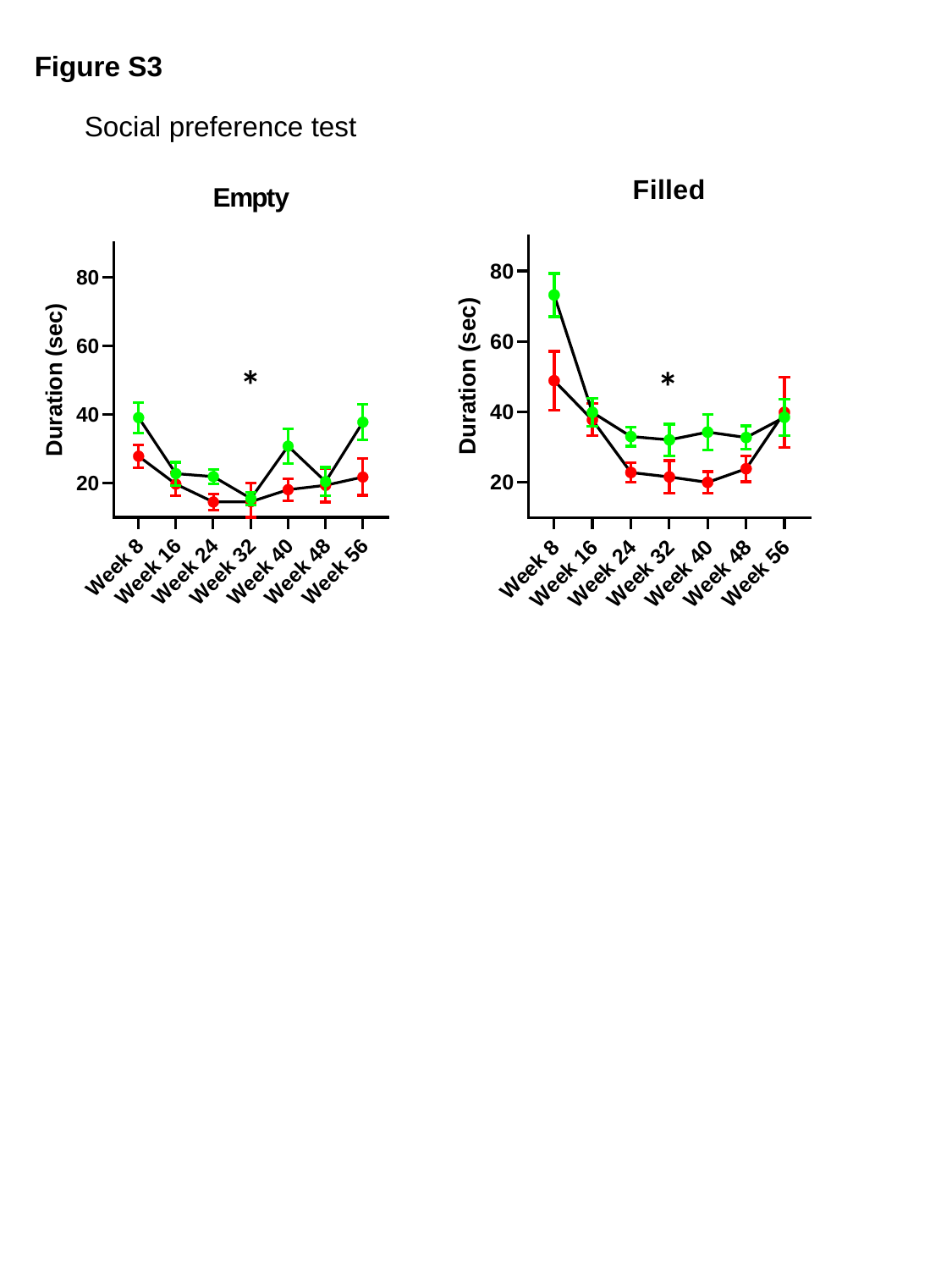

Figure S3
Social preference test
*
*

Supplement: Supplementary Figure 3 — Duration of interaction with the empty and the filled compartment during testing for social preference is shown as means ± SEM for deaf and sham or naïve controls for the weeks of testing. Differences between lesion rats and controls are shown as asterisks (*P < 0.05). [file Presentation_3.PPTX]
